# Contrast-Enhanced Spectral Mammography-Based Prediction of Non-Sentinel Lymph Node Metastasis and Axillary Tumor Burden in Patients With Breast Cancer

**DOI:** 10.3389/fonc.2022.823897

**Published:** 2022-05-06

**Authors:** Xiaoqian Wu, Yu Guo, Yu Sa, Yipeng Song, Xinghua Li, Yongbin Lv, Dong Xing, Yan Sun, Yizi Cong, Hui Yu, Wei Jiang

**Affiliations:** ^1^ Department of Biomedical Engineering, School of Precision Instrument and Opto-Electronics Engineering, Tianjin University, Tianjin, China; ^2^ Department of Radiotherapy, Yantai Yuhuangding Hospital, Yantai, China; ^3^ Department of Radiology, Yantai Yuhuangding Hospital, Yantai, China; ^4^ Department of Otorhinolaryngology–Head and Neck Surgery, Yuhuangding Hospital of Qingdao University, Yantai, China; ^5^ Shandong Provincial Clinical Research Center for Otorhinolaryngologic Diseases, Yantai, China; ^6^ Department of Breast Surgery, Yantai Yuhuangding Hospital, Yantai, China

**Keywords:** breast cancer, radiomics, contrast-enhanced spectral mammography, non-sentinel lymph node metastasis, axillary tumor burden

## Abstract

**Purpose:**

To establish and evaluate non-invasive models for estimating the risk of non-sentinel lymph node (NSLN) metastasis and axillary tumor burden among breast cancer patients with 1–2 positive sentinel lymph nodes (SLNs).

**Materials and Methods:**

Breast cancer patients with 1–2 positive SLNs who underwent axillary lymph node dissection (ALND) and contrast-enhanced spectral mammography (CESM) examination were enrolled between 2018 and 2021. CESM-based radiomics and deep learning features of tumors were extracted. The correlation analysis, least absolute shrinkage and selection operator (LASSO), and analysis of variance (ANOVA) were used for further feature selection. Models based on the selected features and clinical risk factors were constructed with multivariate logistic regression. Finally, two radiomics nomograms were proposed for predicting NSLN metastasis and the probability of high axillary tumor burden.

**Results:**

A total of 182 patients [53.13 years ± 10.03 (standard deviation)] were included. For predicting the NSLN metastasis status, the radiomics nomogram built by 5 selected radiomics features and 3 clinical risk factors including the number of positive SLNs, ratio of positive SLNs, and lymphovascular invasion (LVI), achieved the area under the receiver operating characteristic curve (AUC) of 0.85 [95% confidence interval (CI): 0.71–0.99] in the testing set and 0.82 (95% CI: 0.67–0.97) in the temporal validation cohort. For predicting the high axillary tumor burden, the AUC values of the developed radiomics nomogram are 0.82 (95% CI: 0.66–0.97) in the testing set and 0.77 (95% CI: 0.62–0.93) in the temporal validation cohort.

**Discussion:**

CESM images contain useful information for predicting NSLN metastasis and axillary tumor burden of breast cancer patients. Radiomics can inspire the potential of CESM images to identify lymph node metastasis and improve predictive performance.

## 1 Introduction

The incidence of breast cancer is increasing, and breast cancer has overtaken lung cancer as the world’s leading cancer (1). Whether axillary lymph node metastasis occurs in breast cancer patients is critical for treatment planning and prognostic evaluation. Sentinel lymph node biopsy is a common method to identify the axillary lymph node metastasis status (2). For patients with positive sentinel lymph nodes (SLNs), axillary lymph node dissection (ALND) is usually necessary (3). However, previous studies have proven that, for some breast cancer patients, axillary metastases are limited to the SLNs (4). Thus, these patients may get no therapeutic benefit from ALND and suffer from multiple complications after the surgery (5).

The ACOSOG Z0011 trial demonstrated, for some patients with 1–2 positive SLNs who undergo breast-conserving surgery, ALND is unnecessary (6). The guideline from China Anti-Cancer Association recommends that breast cancer patients with 1–2 positive SLNs who meet the criteria of ACOSOG Z0011 trial can only perform SLN biopsy and avoid ALND (7). However, some breast cancer patients with 1–2 positive SLNs may fall outside Z0011 guideline. For example, approximately 80% of breast cancer patients do not perform the breast-conserving surgery in China (8). For these patients, ALND is necessary in the clinic to achieve accurate axillary lymph node (ALN) staging, which helps future medical decisions and prognosis evaluation (9). Developing a non-invasive and effective prediction model suitable for patients with 1–2 positive SLNs is able to avoid ineffective ALND and achieve personalized cancer management.

Furthermore, after the ACOSOG Z0011 trial, the assessment of lymph node status is no longer limited to axillary metastasis but more focused on the axillary tumor burden that indicates the extent of lymph node involvement (10). If the patient has four or more positive ALNs, that is considered as high axillary tumor burden. The ACOSOG Z0011 trial shows that only 13.7% of breast cancer patients with 1–2 positive SLNs have more than three positive ALNs (11), which means that most breast cancer patients with 1–2 positive SLNs have a low axillary tumor burden. The patients with a low axillary tumor burden would be safe from recurrence without ALND (12). The RxPONDER trial shows that postmenopausal breast cancer patients with 1–3 positive ALNs and recurrence score of 25 or less can avoid adjuvant chemotherapy (13). Therefore, developing a non-invasive predictive method for the axillary tumor burden is also important for the personalized cancer management of breast cancer patients with 1–2 positive SLNs.

Several previous studies have demonstrated the utility of clinical risk factors, such as the number of positive SLNs, ratio of positive SLNs, and lymphovascular invasion (LVI) in the prediction of non-sentinel lymph node (NSLN) metastasis for breast cancer patients with 1–2 positive SLNs (14, 15). In predicting the SLN status in breast cancer patients, researchers evaluated the CancerMath model to estimate the probability of having positive lymph nodes and found that addition of prognostic factors human epidermal growth factor receptor 2 (HER-2) and Ki67 could help in improving the classification performances (16, 17). Nevertheless, the predictive ability of clinical risk factors is limited.

Contrast-enhanced spectral mammography (CESM) uses mammography in combination with contrast agent to increase diagnostic capability through detection of areas of increased vascularization in the breast, being useful to diagnose breast disease, indicate preoperative staging of breast cancer, and evaluate the response to neoadjuvant chemotherapy (18, 19). CESM also increases the detection of breast tumors, especially in dense breasts (20). Massafra et al. (21) proposed an automated expert system for discriminating benign and malignant breast cancer lesions based on radiomics analysis of CESM images. Even in the case of metastatic neoplastic disease, CESM represents a valid method to accurately diagnose (22). However, the features of CESM images in identifying lymph node metastasis are not obvious.

Radiomics captures intratumoral heterogeneity in a non-invasive way by extracting large amounts of image features from radiographic images (23). It is potentially applicable to aid cancer detection, diagnosis, assessment of prognosis, and prediction of response to treatment (24). Radiomics has achieved some encouraging outcomes in predicting lymph node metastasis (25). Mao et al. (26) established a CESM-based radiomics nomogram for the prediction of axillary lymph node metastasis in breast cancer with good performance.

Cong et al. (27) studied the relationship between imaging features and NSLN metastasis in mammography and ultrasound and found that tumor size and the number of positive SLNs, mammographic mass margins, and ultrasonographic vascularity were independent predictors of NSLN metastasis in SLN-positive patients of breast cancer. Based on this clinical research, a radiomics nomogram, incorporating CESM-based radiomics score and several clinical risk factors, is proposed in this study to differentiate the status of NSLN metastasis. Besides, we further studied the non-invasive method for axillary tumor burden estimation and developed a radiomics nomogram for predicting the probability of high axillary tumor burden (>3 positive ALNs) for 1–2 positive SLN patients.

## 2 Materials and Methods

We retrospectively collected the clinical data and CESM images of 1–2 positive SLN patients. A radiomics model, a deep learning model, and the model combining deep learning features and radiomics features were compared in predicting NSLN metastasis. Finally, two radiomics nomograms predicting respectively NSLN metastasis status and the probability of high axillary tumor burden were built and evaluated.

### 2.1 Study Participants

**Figure 1:**
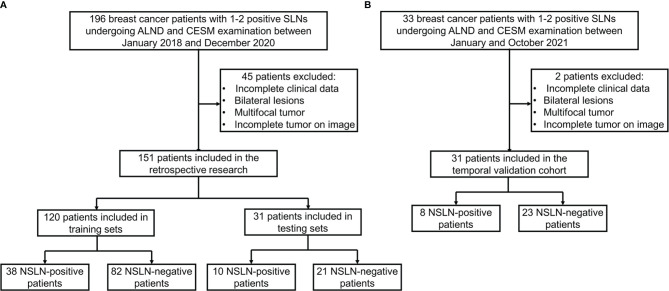
Flow diagram of the study exclusion criteria in the training and testing sets **(A)** and temporal validation cohort **(B)**. CESM, contrast-enhanced spectral mammography; SLN, sentinel lymph node; NSLN, non-sentinel lymph node; ALND, axillary lymph node dissection.

This retrospective study was approved by the ethics committee of Yantai Yuhuangding Hospital. We reviewed 229 breast cancer patients with 1–2 positive SLNs who underwent ALND and CESM examination in the Department of Breast Surgery between January 2018 and October 2021. Incomplete clinical data, bilateral lesions, multifocal tumor, and incomplete tumor on CESM images were excluded from our study. The final dataset included 182 patients, of whom 56 patients were NSLN-positive and 126 patients were NSLN-negative. There are 34 patients with high axillary tumor burden and 148 patients with low axillary tumor burden in the dataset. A total of 151 patients between 2018 and 2020 were split randomly into training and testing sets in a ratio of 8:2. The temporal validation cohort contained 31 patients in 2021. The participant selection is detailed in **Figure 1**.

### 2.2 Sentinel Lymph Node Biopsy

SLN mapping was performed using lymphoscintigraphy with methylene blue dye. On the day of the operation, technetium-99 sulfur colloid (Beijing Shihong Pharmaceutical Development Center, Beijing, China) was injected intradermally above the tumor, peritumorally, or at the areola of the breast. Methylene blue dye (Jumpcan, Taixing, China) was injected 15 min before surgery. During surgery, the SLN was localized by using a γ-probe (Neoprobe Corporation, Dublin, OH, USA). The SLN was defined as a blue lymph node and/or a lymph node with an *ex vivo* radioactive count ≥10% of the *ex vivo* radioactive count of the hottest lymph node; the other axillary lymph nodes were defined as NSLNs.

### 2.3 Pathological Examinations

All axillary lymph nodes including SLNs and NSLNs were subjected to standard evaluation with H&E-stained sections. The nodal tissue was fixed in 10% formalin and embedded in paraffin. After this fixation, serial sections of the lymph nodes were obtained for definitive analysis. Tumor deposits were categorized as isolated tumor cells (≤0.2 mm), micrometastases (0.2–2 mm), or macrometastases (>2 mm). Macrometastases and micrometastases were considered as positive lymph nodes.

### 2.4 Contrast-Enhanced Spectral Mammography Image Acquisition

All patients underwent CESM examination before ALND. CESM images were obtained using the Senographe Essential all-digital mammography system (GE Healthcare, Inc., Princeton, USA), including low-energy and recombined images in Digital Imaging and Communications in Medicine (DICOM) format. After injecting the intravenous iodine contrast agent (1.5 ml/kg body weight, flow rate of 3.0 ml/s), the mammograms including craniocaudal (CC) and mediolateral oblique (MLO) views are obtained around 2 min later, while the breast remains compressed. After low-energy and high-energy exposure, eight images are collected within 5 min. Then, four recombined images are obtained after the subtraction of low-energy and high-energy images for each position on the workstation. Each image was in DICOM format with the image size of 3,062 × 2,394.

### 2.5 Radiomics and Deep Learning Models

**Figure 2:**
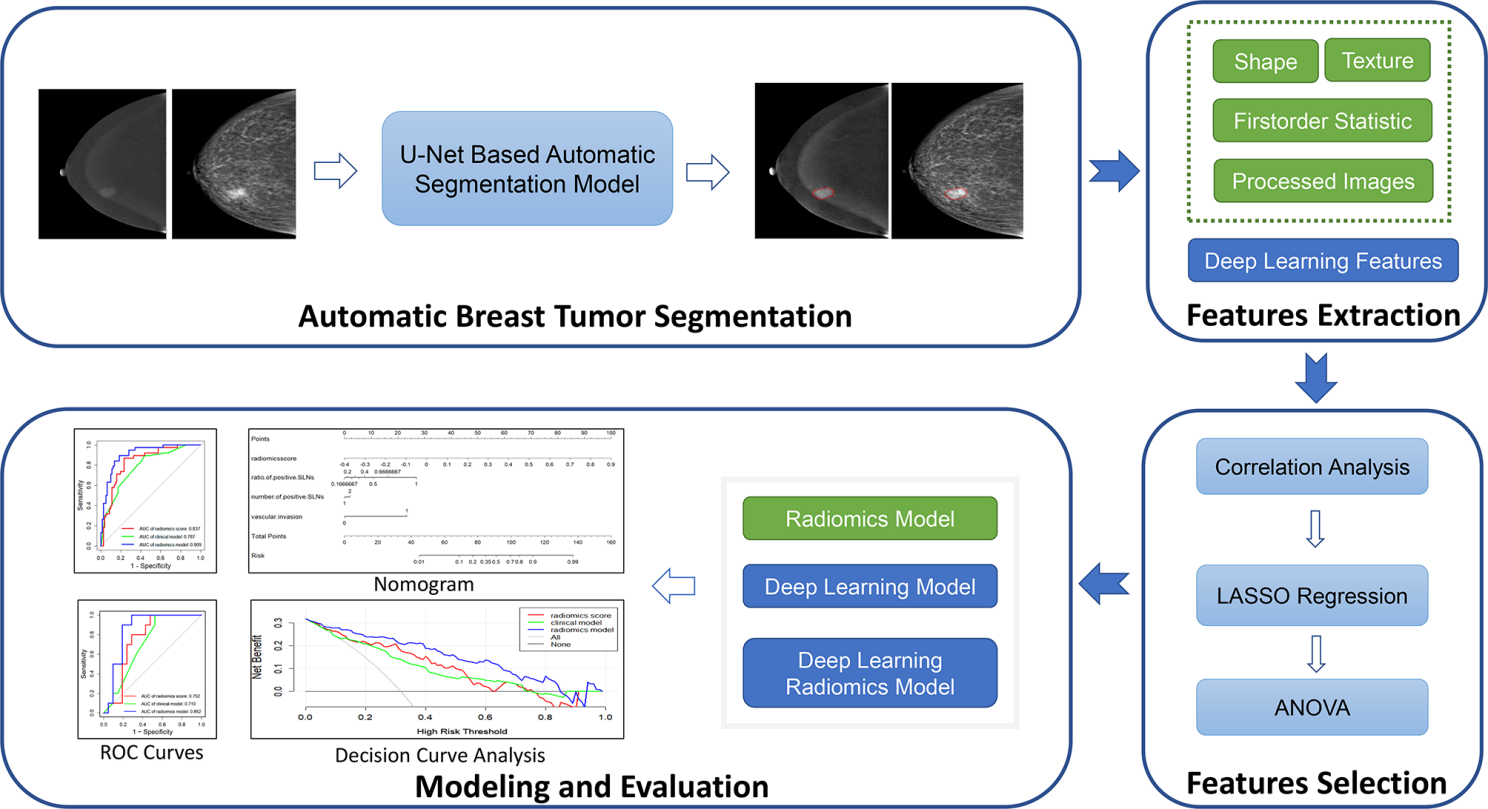
Overview of the construction of the prediction models. LASSO, least absolute shrinkage and selection operator; ANOVA, analysis of variance; ROC, receiver operating characteristic.

The overall workflow of this study is illustrated in **Figure 2**. Firstly, a deep learning-based breast tumor segmentation method was used to automatically delineate breast tumor regions on CESM images with CC and MLO views. CESM image features in tumor regions, including radiomics and deep learning features, are extracted, from which several key features are further selected. Finally, prediction models are developed by combining the selected image features and clinical risk factors. The area under the receiver operating characteristic (ROC) curve (AUC) (28) and decision curve analysis (DCA) (29) are used for evaluating these models. Nomograms are also given to show understandable outcome measures.

#### 2.5.1 Automatic Breast Tumor Segmentation

**Figure 3:**
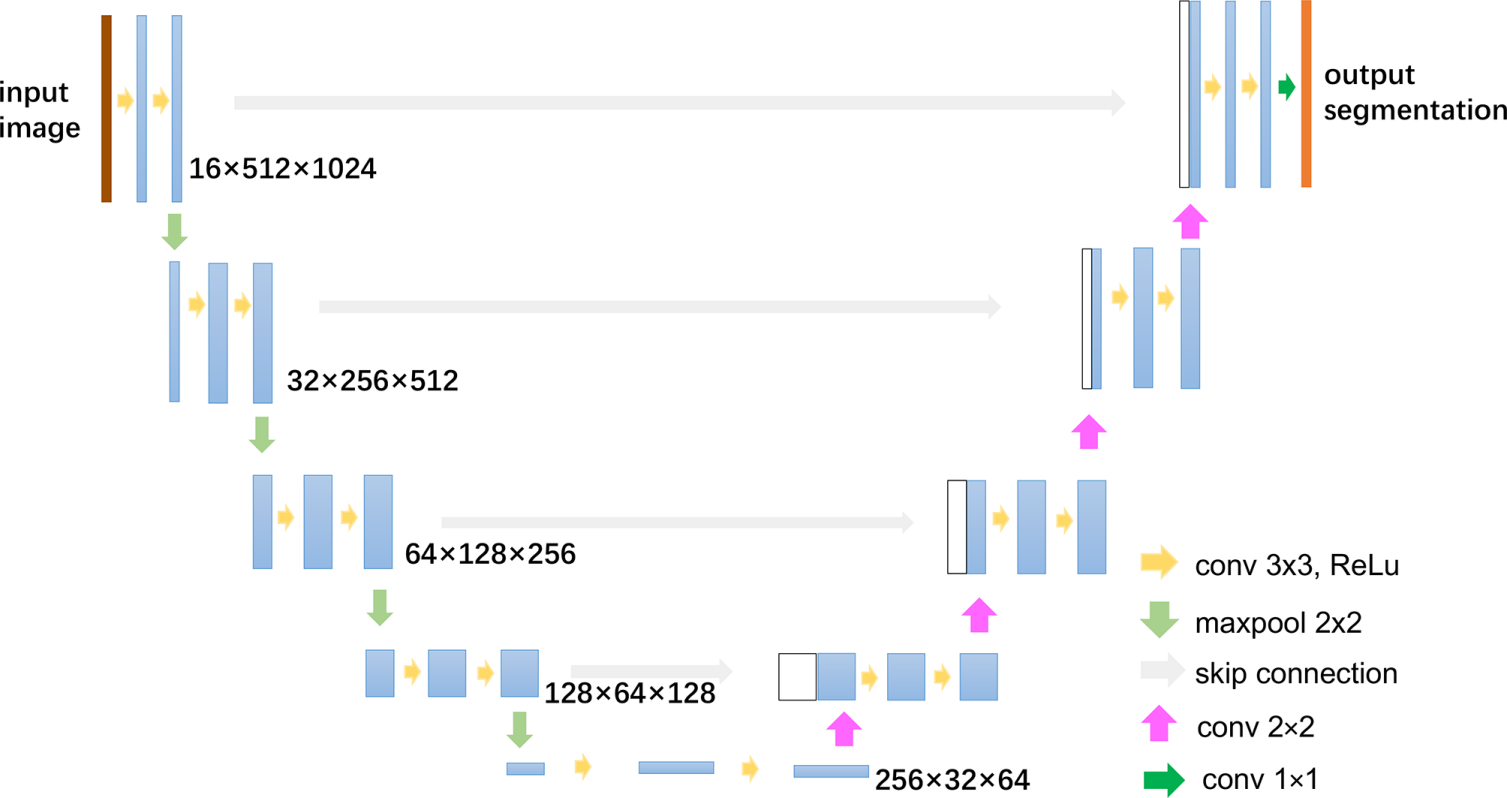
U-Net architecture.

The automatic segmentation of breast cancer tumors was carried out by U-Net, a commonly used deep learning-based medical image segmentation method, which has achieved good performance in lots of medical image segmentation tasks (30, 31). The architecture and parameters of U-Net is shown in **Figure 3**. The low-energy and recombined images with the same view were used as the input of the network. Before training, the gray-level range of each image was adjusted *via* the self-adaptive contrast enhancement. Then, the intensity scale was normalized to (0,1) by max–min normalization as follows:


(1) $$X_{norm}=\frac{X-X_{min}}{X_{max}-X_{min}}$$

where *X_norm_* was the normalized gray matrix, the *X* was the gray matrix of the original image, *X_min_* denoted the minimum gray value, and *X_max_* was the maximum gray value.

In order to augment and increase the training dataset, we applied the horizontal flip, rotation in a range of ±10 degrees, horizontal and vertical offset by 10%, and zoom in and out by 10%. The size of the augmented dataset is 10 times larger than the original training dataset. The U-Net loss function *L_total_* was determined as the sum of the dice loss *L_Dice_* and the cross-entropy loss *L_CE_* (32). The hyperparameter learning rate of the optimizer was set to 0.01. The batch size was set to 4, and training was conducted for 100 epochs.

As the U-Net is a supervised segmentation method, label data are needed to train the network. In this study, the labels of tumor regions of interest (ROIs) were manually delineated by two experienced breast radiologists (one with 7 years of experience in breast imaging, and another with 10 years of experience in breast imaging) blinded to pathological outcomes in CC and MLO images *via* the MIM software (version 6.8.2, MIM Software Inc., Cleveland, OH, USA). All disagreements were resolved by a senior breast radiologist with 15 years of experience in breast imaging. All the CESM images in our dataset have manual tumor delineations, which are used not only to train the segmentation network but also to evaluate the segmentation performance.

#### 2.5.2 Feature Extraction

Two groups of image features were extracted. The first group contains radiomics features defined by the Imaging Biomarker Standardization Initiative (33) including shape, first-order statistics, and texture features. We used logarithm, square root, square, and exponential transformation to enhance image contrast, wavelet transform decomposing image signal into different subbands to enhance the details of images, and gradient transformation to highlight the images’ edge information. Radiomics features were extracted not only from the ROIs in original CESM images but also from these processed images. A total of 3,738 features were extracted, as shown in **Supplementary Figure S1**. Open-source python package pyradiomics v3.0.1 (34) was used for the above radiomics feature extraction.

The second group was composed of deep learning features extracted by pretrained ResNet-18 network (35). Deep learning networks have been shown as powerful classifiers and can automatically extract multilevel abstract and discriminative features from big data sets. Even though deep learning algorithms have been improving, few data are still a critical factor limiting the learning of complex tasks. Transfer learning is a popular approach for improving classification performance when image data are limited, especially in the medical field (36, 37). The pretrained models in natural image databases such as ImageNet are beneficial to train deep learning models for medical image classification (38). The popular networks for transfer learning include ResNet, VGG, and AlexNet. ResNet with the residual blocks not only solves the degradation problem of deep layer networks but also needs fewer parameters compared to the traditional convolutional neural network (CNN). It always shows higher precision in classification (39).

Here, ResNet-18 network-based transfer learning is used to extract CESM deep learning features. The network structure of ResNet-18 was shown in **Supplementary Figure S2**. Images containing only the tumor ROIs of CESM images were resized to 224 × 224 with bilinear interpolation and input into the pretrained ResNet-18 network. The penultimate fully connected layer output with the length of 512 was used as the deep learning feature group. For each patient, the deep learning features were extracted from low-energy and recombined images in CC and MLO views.

#### 2.5.3 Feature Selection and Radiomics Score Development

After performing Z-score normalization on the extracted features so that the mean value of each normalized feature vector was 0 and the standard deviation was 1, the correlation analysis was first used to eliminate redundant features, which have a high correlation with other features (the absolute values of correlation coefficients greater than 0.85). Then, a least absolute shrinkage and selection operator (LASSO) regression (40) model was fit on the training set. The optimal LASSO alpha parameter was set by 10-fold cross-validation, and the features with non-zero coefficients were reserved. We also used analysis of variance (ANOVA) (41) to further select the features that had significant differences (P < 0.05) between different patient groups (for example, NSLN-positive and NSLN-negative patient groups). Finally, a radiomics score, a deep learning score, and a deep learning radiomics score were built by linearly combining respectively the radiomics features, the deep learning features, and the deep learning radiomics features. The correlation analysis, LASSO regression, and ANOVA methods were performed by “python” scikit-learning and pandas package.

#### 2.5.4 Construction and Validation of the Radiomics Model

Previous studies have proven that the combination of clinical factors and radiomics score performed better in terms of disease diagnosis (42). In our study, one-way ANOVA was used to select the clinical risk factors related to the final prediction results. Models incorporating the above three radiomics score and the selected clinical risk factors were consequently developed by training a multivariable logistic regression in the training set.

The variance inflation factor (43) was used to access the multicollinearity in our regression models. A variance inflation factor lower than 10 means no multicollinearity. Besides, the good fitness for logistic regression was evaluated by the Hosmer–Lemeshow test (44). ROC curves were applied to measure the prediction accuracy of different models. The optimal threshold values (cutoff points) were determined by maximizing the Youden index, and the AUC, accuracy, sensitivity, specificity, positive predictive value (PPV), and negative predictive value (NPV) of different models were calculated. The clinical utility of the proposed models was also evaluated by DCA.

### 2.6 Statistical Analysis

Categorical variables were compared using the chi-square test or Fisher’s exact test, while continuous variables were compared using t-test. DeLong test (45) was used to compare the AUC difference between different models. P values <0.05 were regarded as a statistically significant difference. The statistical analysis was performed with SPSS (version 25.0, www.ibm.com/products/spss-statistics) and R software (version 4.0.5, R Project for Statistical Computing, www.r-project.org). The main R packages used in this study included rms, pROC, rmda, PredictABEL, and ggplot2.

## 3 Results

### 3.1 Clinical Characteristics

There are 120 patients in the training group, 31 patients in the testing group, and 31 patients in the temporal validation cohort. The clinical characteristics of these patients are shown in **Table 1**. Significant differences were found in the number of positive SLNs (P = 0.008), the ratio of positive SLNs (P < 0.001), and LVI (P < 0.001) between NSLN-negative and NSLN-positive patients in the training set. The rates of NSLN metastasis were 31.7% (38 of 120), 32.2% (10 of 31), and 25.8% (8 of 31) in the training set, testing set, and temporal validation cohort, respectively.

**Table 1:** Patients’ clinical characteristics.

Characteristic	Training set (N = 120)		P	Testing set (N = 31)		P	Temporal Validation cohort (N = 31)		P
	Negative NSLNs	Positive NSLNs		Negative NSLNs	Positive NSLNs		Negative NSLNs	Positive NSLNs	
Age (years), (mean ± SD), years	55.30 ± 10.15	53.89 ± 10.19	0.483	56.10 ± 10.31	54.80 ± 4.87	0.638	54.70 ± 11.66	58.25 ± 7.61	0.430
Pathology type			0.590			–			0.520
Ductal breast cancer	75	35		21	10		20	7	
Lobular breast cancer	5	3		0	0		1	1	
Others	2	0		0	0		2	0	
Histological grade			0.294			0.170			0.170
1	14	2		3	0		2	0	
2	50	25		12	4		18	4	
3	13	9		6	6		2	3	
NA	5	2		0	0		1	1	
Number of positive SLNs			0.008			0.213			0.002
1	61	19		17	6		21	3	
2	21	19		4	4		2	5	
Number of positive axillary lymph nodes			–			–			–
≤3	82	16		21	1		23	5	
>3	0	22		0	9		0	3	
Ratio of positive SLNs, (mean ± SD)	0.58 ± 0.30	0.78 ± 0.27	<0.001	0.64 ± 0.30	0.82 ± 0.24	0.115	0.41 ± 0.22	0.61 ± 0.21	0.033
ER status			0.249			0.109			
Negative	4	4		3	4		1	2	0.089
Positive	78	34		18	6		22	6	
PR status			0.725			0.525			0.236
Negative	7	4		6	4		2	2	
Positive	75	34		15	6		21	6	
HER-2 status			0.458			0.034			0.282
Negative	73	32		18	5		20	8	
Positive	9	6		3	5		3	0	
Ki67			1.000			0.093			0.746
<14%	41	19		6	6		4	1	
≥14%	41	19		15	4		19	7	
LVI			<0.001			0.353			0.031
Negative	73	22		16	6		20	4	
Positive	9	16		5	4		3	4	

SLN, sentinel lymph node; NSLN, non-sentinel lymph node; ER, estrogen receptor; PR, progesterone receptor; HER-2, human epidermal growth factor receptor 2; LVI, lymphovascular invasion.

### 3.2 Automatic Breast Tumor Segmentation Performance

**Figure 4:**
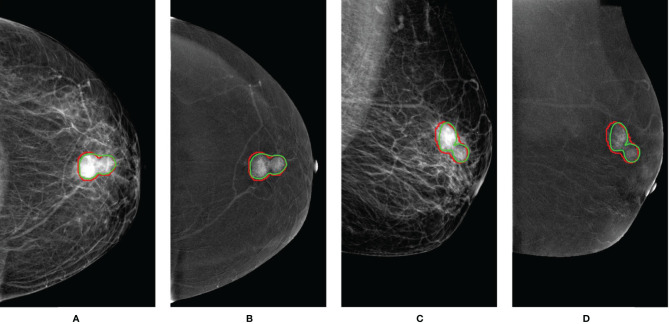
An example of breast tumor segmentation. The green lines are automatic segmentation results. The red lines are manual delineations of tumors. For images with CC views **(A, B)**, the Dice score is 0.91. For images with MLO views **(C, D)**, the Dice score is 0.85. CC, craniocaudal; MLO, mediolateral oblique.

For automatic breast tumor segmentation, the patients with incomplete clinical data but high CESM image quality were also included in the segmentation dataset, which contains a total of 197 patients’ CESM images. This dataset was split randomly into the training (n = 177) and testing sets (n = 20). A 5-fold cross-validation was adopted for U-Net training. The segmentation performance was evaluated with the Dice score, and the mean Dice score of the proposed segmentation method is 0.84 ± 0.10 in the testing set. Automatic breast tumor segmentation results of a patient are shown in **Figure 4**. The automatic tumor segmentation results are close to the manual delineation and show good segmentation accuracy.

### 3.3 Prediction Performance of Radiomics Model for Non-Sentinel Lymph Node Metastasis Status

#### 3.3.1 Feature Selection and Radiomics Score Development

**Figure 5:**
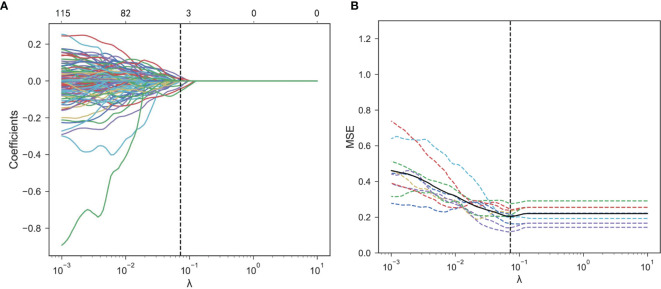
Radiomics feature selection using the LASSO logistic regression. **(A)** Mean square error (MSE) path using 10-fold cross-validation. The dotted vertical line means that the optimal value of λ was 0.072. **(B)** LASSO coefficient profiles of the 368 features. Six features with non-zero coefficients were selected at a λ value of 0.072. LASSO, least absolute shrinkage and selection operator.

Feature selections were performed respectively in the radiomics feature group, the deep learning feature group, and the deep learning radiomics feature group composed of radiomics features and deep learning features. The correlation analysis selected 368 radiomics features and 2,048 deep learning features because deep learning features have low correlation with each other. After LASSO logistic regression, 6 radiomics features, 137 deep learning features, and 8 deep learning radiomics features with non-zero coefficients were selected in the three feature groups. **Figures 5A, B** show the radiomics feature selection of parameter λ. Finally, ANOVA reserved 5 radiomics features, 61 deep learning features, and 6 deep learning radiomics features. Based on the three feature selection results, radiomics score, deep learning score, and deep learning radiomics score were constructed *via* linear combinations of the selected features in different feature groups. The NSLN metastasis status prediction performances of the radiomics score, deep learning score, and deep learning radiomics score are shown in **Table 2**. The deep learning radiomics score and the radiomics score performed better in the testing dataset when compared to the deep learning score, which has the best training AUC value but a poor testing AUC value, owing to the overfitting of the model.

**Table 2:** Summary of the performance of different radiomics scores.

	AUC of Training Set (95% CI)	P	AUC of Testing Set (95% CI)	P
Radiomics Score	0.84 (0.76–0.91)	0.402	0.74 (0.56–0.92)	0.805
Radiomics Model	0.91 (0.86–0.97)	0.001	0.85 (0.71–0.99)	0.046
Deep Learning Score	1.0 (1.0–1.0)	<0.001	0.44 (0.22–0.65)	0.109
Deep Learning Model	1.0 (1.0–1.0)	<0.001	0.53 (0.31–0.75)	0.121
Deep Learning Radiomics Score	0.84 (0.77–0.93)	0.385	0.76 (0.59–0.93)	0.596
Deep Learning Radiomics Model	0.83 (0.76–0.91)	0.070	0.73 (0.52–0.94)	0.821

CI, confidence interval; P value, compared to the clinical model.

#### 3.3.2 Construction of the Radiomics Model

In one-way ANOVA, the number of positive SLNs (P = 0.008), the ratio of positive SLNs (P < 0.001), and LVI (P < 0.001) were proven as effective predictors for identifying the NSLN metastasis. By combining these clinical risk factors respectively with the radiomics score, deep learning score, and deep learning radiomics score, the radiomics model, the deep learning model, and the deep learning radiomics model were built using multivariate logistic regression. The radiomics model showed significantly better performance than that of the deep learning model and the deep learning radiomics model and achieved an AUC value of 0.85 [95% confidence interval (CI): 0.71–0.99] in the testing set, as shown in **Table 2**. The radiomics model was finally proposed in this study to predict NSLN metastasis status due to its good prediction performance (P = 0.046 compared to the clinical model).

**Figure 6:**
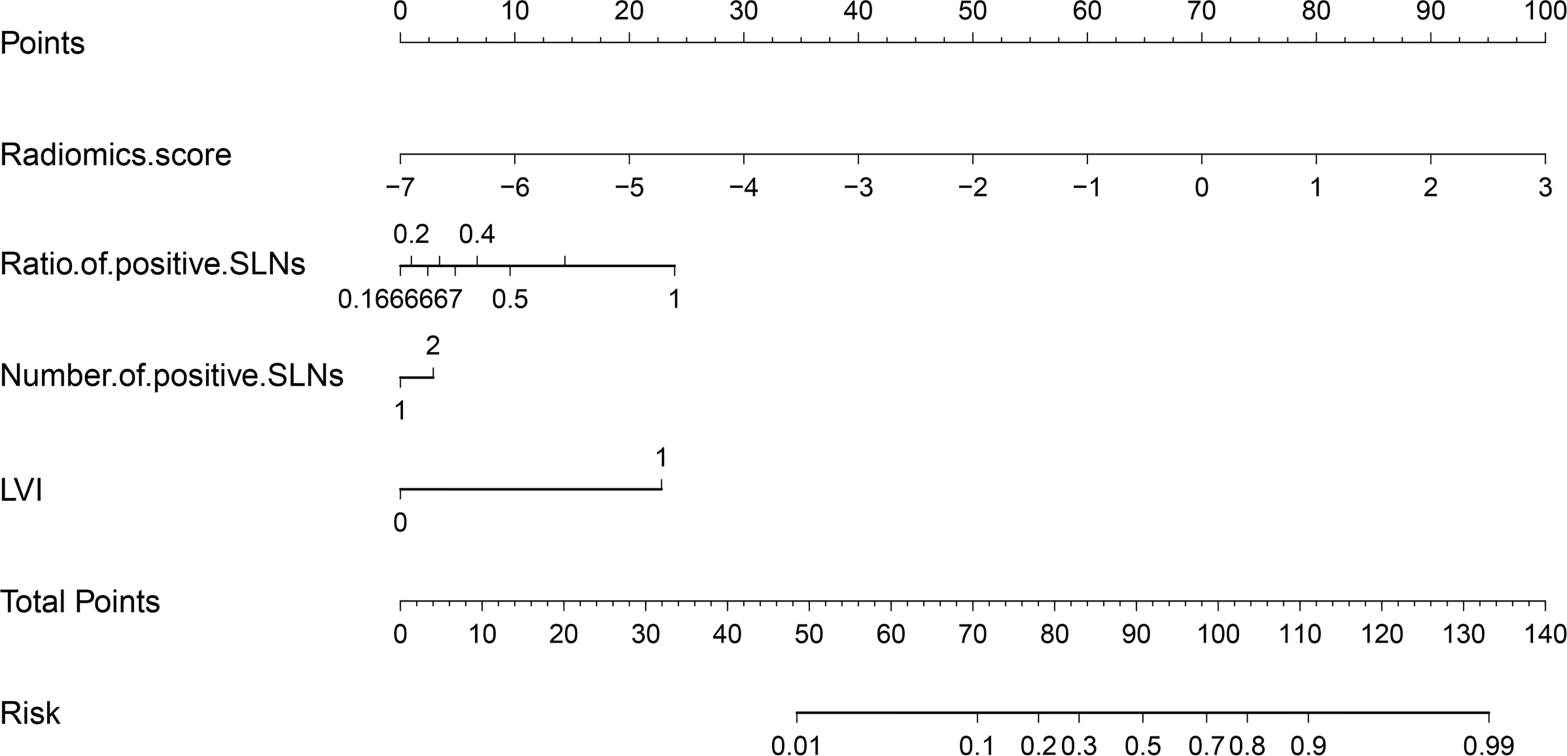
Radiomics nomogram to predict NSLN metastasis. (50% probability is used as the classification cutoff point, corresponding to 91 points). SLN, sentinel lymph node; LVI, lymphovascular invasion.

Based on the radiomics model, an understandable and visual nomogram was also constructed for more convenient clinical application, as shown in **Figure 6**. The calibration plot for the nomogram is shown in **Supplementary Figure S3**. The regression coefficients of the radiomics score and radiomics model are shown in **Table 3**. The variance inflation factors of the four predictors used in the radiomics nomogram (radiomics score, the number of positive SLNs, the ratio of positive SLNs, and LVI) ranged from 1.03 to 1.15, which means no multicollinearity.

**Table 3:** The corresponding coefficients for establishing the radiomics score (A) and radiomics model (B).

	Coefficient	Odds ratio (95% CI)	P
**(A)**			
Interception	-2.23		0.788
Low-Energy_CC_wavelet-LH_GLCM_IMC1^*^	1.88	6.57 (0.93–46.43)	0.059
Low-Energy_MLO_wavelet-HH_firstorder_Median^*^	-0.09	0.91 (0.86–0.97)	0.003
Low-Energy_MLO_logarithm_NGTDM_Contrast^*^	-3.41	0.03 (0.00–0.26)	0.001
Recombined_CC_waveletLH_GLSZM_GrayLevelNonUniformityNormalized^*^	0.43	1.54 (1.13–2.10)	0.007
Recombined_CC_exponential_GLDM_DependenceVariance	0.59	1.81 (0.98–3.33)	0.059
**(B)**			
Interception	-2.78		<0.001
Radiomics score	1.09	2.96 (1.87–4.69)	<0.001
Number of positive SLNs	0.31	1.37 (0.43–4.38)	0.600
Ratio of positive SLNs	3.12	22.70 (2.92–176.46)	0.003
LVI	2.48	11.91(2.99–47.47)	0.004

Features with * need to be multiplied by 100.

CI, confidence interval; SLN, sentinel lymph node; LVI, lymphovascular invasion.

**Figure 7:**
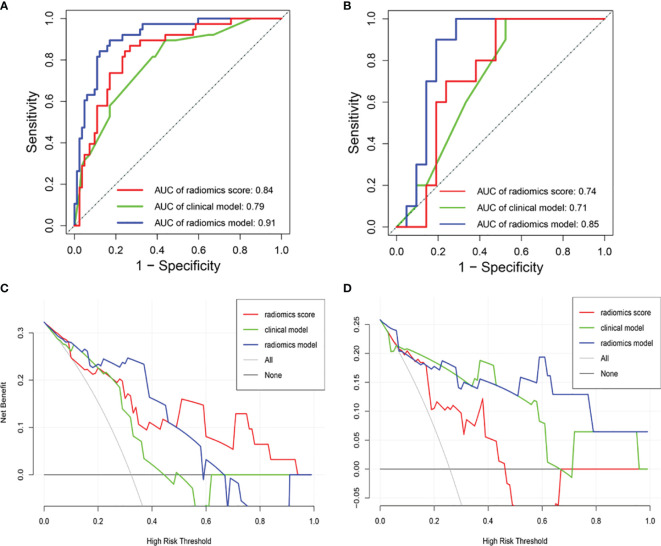
Receiver operating characteristic (ROC) curves of the radiomics score, clinical model, and radiomics model in the **(A)** training and **(B)** testing sets. DCA of the three models in **(C)** the testing set and **(D)** the temporal validation set. The y-axis measures the net benefit. The blue line means the radiomics score. The green line means the clinical model. The red line means the radiomics model. The horizontal black thin line means the assumption that all breast cancer patients were NSLN-positive. The gray line means the assumption that all patients were NSLN-negative. DCA, decision curve analysis.

**Figures 7A, B** show the ROCs of the radiomics score, clinical model, and the proposed radiomics model for predicting NSLN metastasis. AUC values of these models were 0.74 (95% CI: 0.56–0.92), 0.71 (95% CI: 0.53–0.89), and 0.85 (95% CI: 0.71-0.99) in the testing set, respectively. DeLong test shows that there are significant differences between the radiomics score and radiomics model (P = 0.004) and between the clinical model and the radiomics model (P = 0.001) in the training set and between the clinical model and the radiomics model (P = 0.046) in the testing set. Furthermore, in the temporal validation cohort, the radiomics model achieved an AUC of 0.82 (95% CI: 0.67–0.97) and an accuracy of 74% (95% CI: 0.55–0.0.88) but showed no difference compared to the clinical model. The prediction performances of the radiomics model incorporating the radiomics score and the clinical risk factors are shown in **Table 4A**.

**Table 4:** Predictive performances of different models.

		Accuracy (95% CI)	Sensitivity (95% CI)	Specificity (95% CI)	PPV (95% CI)	NPV (95% CI)
**(A)**
Radiomics Score	Training set	0.78 (0.69–0.85)	0.87 (0.71–0.95)	0.73 (0.62–0.82)	0.60 (0.46–0.73)	0.92 (0.82–0.97)
	Testing set	0.68 (0.49–0.83)	1.00 (0.66–1.00)	0.52 (0.30–0.74)	0.50 (0.28–0.72)	1.00 (0.68–1.00)
	Temporal validation cohort	0.71 (0.52–0.86)	0.88 (0.47–0.99)	0.65 (0.43–0.83)	0.47 (0.22–0.73)	0.94 (0.67–1.00)
Clinical Model	Training set	0.67 (0.57–0.75)	0.90 (0.74–0.97)	0.56 (0.45–0.67)	0.49 (0.37–0.61)	0.92 (0.80–0.97)
	Testing set	0.65 (0.45–0.81)	1.00 (0.66–1.00)	0.48 (0.26–0.70)	0.48 (0.26–0.70)	1.00 (0.66–1.00)
	Temporal validation cohort	0.77 (0.59–0.90)	0.88 (0.47–0.99)	0.74 (0.51–0.89)	0.54 (0.26–0.80)	0.94 (0.71–1.00)
Radiomics Model	Training set	0.85 (0.77–0.91)	0.89 (0.74–0.97)	0.83 (0.73–0.90)	0.71 (0.56–0.83)	0.94 (0.86–0.98)
	Testing set	0.81 (0.63–0.93)	1.00 (0.66–1.00)	0.71 (0.48–0.88)	0.63 (0.36–0.84)	1.00 (0.75–1.00)
	Temporal validation cohort	0.74 (0.55–0.88)	1.00 (0.60–1.00)	0.65 (0.43–0.83)	0.50 (0.26–0.74)	1.00 (0.75–1.00)
**(B)**						
Radiomics Score	Training set	0.79 (0.71–0.86)	0.96 (0.76–1.00)	0.76 (0.66–0.83)	0.48 (0.33–0.63)	0.99 (0.92–1.00)
	Testing set	0.68 (0.49–0.83)	0.89 (0.51–0.99)	0.60 (0.37–0.79)	0.47 (0.24–0.71)	0.93 (0.64–1.00)
	Temporal validation cohort	0.61 (0.42–0.78)	1.00 (0.31–1.00)	0.57 (0.37–0.75)	0.20 (0.05–0.49)	1.00 (0.76–1.00)
Clinical Model	Training set	0.64 (0.55–0.73)	0.86 (0.64–0.96)	0.59 (0.49–0.69)	0.32 (0.21–0.46)	0.95 (0.85–0.99)
	Testing set	0.65 (0.45–0.81)	0.78 (0.40–0.96)	0.59 (0.37–0.79)	0.44 (0.21–0.70)	0.87 (0.58–0.98)
	Temporal validation cohort	0.87 (0.70–0.96)	1.00 (0.31–1.00)	0.86 (0.66–0.95)	0.43 (0.12–0.80)	1.00 (0.83–1.00)
Radiomics Model	Training set	0.79 (0.71–0.86)	1.00 (0.82–1.00)	0.74 (0.65–0.83)	0.47 (0.32–0.62)	1.00 (0.94–1.00)
	Testing set	0.75 (0.55–0.88)	0.88 (0.51–0.99)	0.68 (0.45–0.85)	0.53 (0.27–0.77)	0.94 (0.68–1.00)
	Temporal validation cohort	0.74 (0.55–0.88)	1.00 (0.31–1.00)	0.71 (0.51–0.86)	0.27 (0.07–0.61)	1.00 (0.80–1.00)

CI, confidence interval; NPV, negative predictive value; PPV, positive predictive value.

(A) Models for identifying NSLN metastasis. (B) Models for predicting high axillary tumor burden.

DCA shows that the radiomics model could add more net benefits than “all treatment” or “none treatment” with the threshold probability range from 0 to 0.65 in the testing set and from 0 to 0.1 and 0.5 to 1.0 in the temporal validation set, as shown in **Figures 7C, D**. The net benefit was calculated as the theoretical relationship between the threshold probability and the relative values of false-positive and false-negative results. The Hosmer–Lemeshow test shows that the radiomics model was no deviation from the perfect fit (P = 0.484).

In our research, 5 useful radiomics features were selected from the CESM image features to develop the radiomics score for NSLN metastasis status prediction, 3 features from the low-energy image, and 2 features from the recombined image. The proposed radiomics model is available on Github^1^. The heatmap in **Supplementary Figure S4** shows the quantitative difference of the 5 selected radiomics features between NSLN-negative and NSLN-positive patients. **Table 5A** presents that the “NGTDM-Contrast” feature and “GLSZM-GrayLevelNonUniformityNormalized” feature have a strong correlation with patients’ NSLN metastasis status, which is consistent with the study from Dong et al. (46).

**Table 5:** Spearman rank correlation between selected features and prediction results.

Image Type	Position	Feature	*r_s_*	P
**(A)**				
Low-energy image	CC	wavelet-LH_GLCM_IMC1	0.20	0.015
	MLO	wavelet-HH_Firstorder_Median	-0.33	<0.001
	MLO	logarithm_NGTDM_Contrast	-0.24	0.003
Recombined image	CC	wavelet-LH_GLSZM_GrayLevelNonUniformityNormalized	0.24	0.003
	CC	exponential_GLDM_DependenceVariance	0.10	0.203
**(B)**				
Low-energy image	MLO	Wavelet-HH-firstorder-Median	-0.23	0.004
	MLO	Wavelet-HH-firstorder-Skewness	0.18	0.025
	MLO	wavelet-HH_GLCM_MCC	0.20	0.783
	MLO	logarithm-NGTDM-Contrast	-0.23	0.005
Recombined image	CC	Original-GLRLM-LongRunLowGrayLevelEmphasis	-0.16	0.048
	CC	Wavelet-LH-GLSZM-GrayLevelNonUniformityNormalized	0.22	0.007
	CC	wavelet-HH_firstorder_Kurtosis	-0.12	0.145
	MLO	original_firstorder_10Percentile	0.20	0.013
	MLO	Original-firstorder-Skewness	-0.30	<0.001
	MLO	logarithm_glrlm_ShortRunLowGrayLevelEmphasis	-0.22	0.007
	MLO	logarithm-GLSZM-GrayLevelNonUniformityNormalized	0.15	0.070
	MLO	logarithm-GLSZM-LargeAreaEmphasis	0.10	0.223

Informational Measure of Correlation 1 (IMC1): the complexity of the texture by using mutual information.

Median: the median gray-level intensity within the ROI.

Contrast: the measure of spatial intensity change.

GrayLevelNonUniformityNormalized: the variability of gray-level intensity values in the recombined image, with a lower value indicating a greater similarity in intensity values.

DependenceVariance: the variance in dependence size in the image.

The underlined features are not only related with the NSLN metastasis but also associated with axillary tumor burden.

(A) For NSLN metastasis status prediction. (B) For high axillary tumor burden prediction.

### 3.4 Performance of the Radiomics Model for Axillary Tumor Burden Prediction

Because of the good performance of the radiomics model on the prediction of NSLN metastasis status, we used the same methods to develop a model for axillary tumor burden prediction.

**Figure 8:**
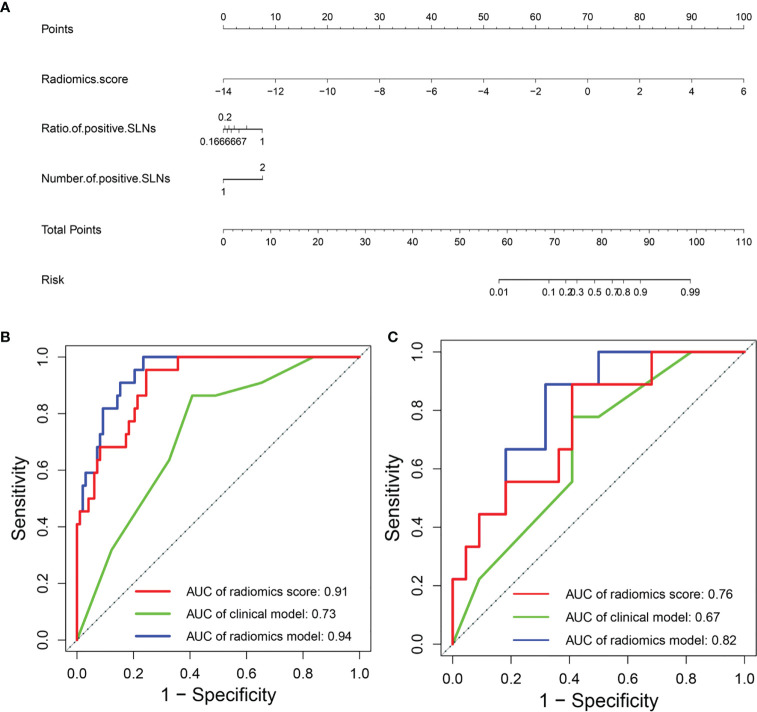
**(A)** Radiomics nomogram to predict the probability of high axillary tumor burden. ROC curves of the clinical model and radiomics model in the **(B)** training and **(C)** testing sets. ROC, receiver operating characteristic.

For predicting high axillary tumor burden, LASSO regression selected 27 features from 368 features, and ANOVA further reserved 12 features, based on which the radiomics score for predicting the axillary tumor burden was calculated. Furthermore, the number of positive SLNs (P = 0.019) and the ratio of positive SLNs (P = 0.001) were the clinical risk factors related to the occurrence of more than 3 positive SLNs according to one-way ANOVA. The radiomics nomogram using patients’ radiomics scores and clinical risk factors to predict the probability of high axillary tumor burden is shown in **Figure 8A**. The variance inflation factors of the three predictors (radiomics score, number of positive SLNs, and ratio of positive SLNs) ranged from 1.04 to 1.25. **Figures 8B, C** show the ROCs of different prediction models. For the testing set, AUC values of the radiomics score, clinical model, and radiomics model were 0.76 (95% CI: 0.57–0.95), 0.67 (95% CI: 0.47–0.87), and 0.82 (95% CI: 0.67–0.97), respectively. In the temporal validation cohort, the AUC of radiomics model was 0.77 (95% CI: 0.62–0.93). DeLong test shows that there are significant differences between the clinical model and radiomics model (P < 0.001) and between the radiomics score and radiomics model (P = 0.049) in the training set, but there is no significant difference between the different models in the testing and temporal validation set.

**Table 4B** summarized the prediction performance of different models, and the radiomics model outperformed the other models with a prediction accuracy of 79% (95% CI: 0.71–0.86) in the training set, 75% (95% CI: 0.55–0.88) in the testing set, and 74% (95% CI: 0.55–0.88) in the temporal validation cohort.

We also explored the Spearman’s rank correlation between 12 radiomics features and axillary tumor burden as shown in **Table 5B**. Most of the selected radiomics features have a strong correlation with patients’ axillary tumor burden. CESM-based radiomics features can be used as a significant supplement to non-invasively identify axillary tumor burden in breast cancer, assisting clinicians in determining the best treatment plan for 1–2 positive SLN breast cancer patients.

## 4 Discussion

In this study, we compared the performance of three models, including the radiomics model, deep learning model, and deep learning radiomics model, in predicting NSLN metastasis. In identifying NSLN-negative and NSLN-positive patients before ALND, the CESM-based radiomics model performed well with AUC values of 0.85 in the testing set and 0.82 in the temporal validation cohort, which was better than the other two models.

Accurately identifying whether a breast cancer patient with 1–2 positive SLNs has NSLN metastasis without ALND is important for further treatment and reducing the pain of patients (47). Zheng et al. (14) reviewed 119 breast cancer patients, analyzed the clinical predictive factors, including the invasive tumor size, histological grade, LVI, and overexpression of HER-2, for predicting NSLN metastasis in breast cancer patients with 1–2 positive SLNs, and developed a logistic regression model, yielding the best AUC of 0.71. In this study, the prediction accuracy can be improved by adding the radiomics features into the prediction model.

We also proposed a radiomics model to predict the probability of high axillary tumor burden, which outperformed the radiomics score and clinical model (AUC of 0.82 and 0.76 and 0.67 in the testing set, respectively). In the temporal validation cohort, the radiomics model also demonstrated the AUC value of 0.77 for predicting the probability of high axillary tumor burden. Previous studies took advantage of axillary ultrasound to identify axillary metastasis preoperatively for breast cancer patients (10, 48). However, axillary ultrasound does not accurately differentiate between low and high axillary tumor burden (49). As shown in our results, the CESM-based radiomics model may achieve good axillary tumor burden prediction, guiding individual treatment and the evaluation of clinical curative effect.

CESM is a new and reliable imaging technique. The recombined images in CESM obtained through subtracting high-energy from low-energy images emphasize breast areas with greater angiogenesis (22). The enhanced lesion in the recombined image can provide more detailed information, if the low-energy images did not show any suspicious lesions, playing a key role in supplementary screening (50). This new technique also shows the potential in identifying axillary lymph node metastases of occult breast cancer (51).

Deep learning has shown superior classification accuracy. However, it requires a huge amount of data for network training. Due to the lack of training data, many medical image-related tasks have applied transfer learning to improve classification performance (39). Guo et al. (52) used ultrasound images and a fine-tuned deep learning radiomics model to identify the risk of NSLN involvement in primary breast cancer, implying the promising potential of the deep learning radiomics model in assessing the risk of ALN metastasis. We also used the pretrained ResNet-18 to extract CESM image features. However, the overall performance of the deep learning model declined in the testing set due to overfitting. The combination of deep learning features with radiomics features and clinical risk factors did not improve the prediction accuracy. On the other hand, radiomics aims to extract as many quantitative features as possible from medical images. The radiomics model combining predefined radiomics features with other clinical data has the potential to increase prediction accuracy (24, 41).

To decrease the man-made factor, the U-Net architecture was used for accomplishing automatic breast tumor segmentation. The mean Dice score of automatic segmentation results in the testing set is 0.84, and the segmentation results are close to the manual segmentation of the radiologists. However, the segmentation accuracy is not good enough. The increasing number of CESM images in the training set or developing more robust segmentation algorithms will further improve the accuracy of breast tumor segmentation.

Our retrospective and single-institutional study still had several limitations. First, as the patients in this study were enrolled from a single institution and the patient inclusion criteria were rigorous, the few data limited the performance of the deep learning model in predicting NSLN metastasis. More images and fine-tuning pretrained deep learning networks might improve the predictive performance. Furthermore, other machine learning methods, such as support vector machine and CNN, were not compared with our model because of the training overfitting of these models caused by few data. Future studies should include a highly standardized, large, balanced, and multicenter dataset across patients and institutions. Moreover, the combination with multimodality medical images such as multiparametric breast MRI might further improve the predictive accuracy. The biological meaning of selected radiomics features is yet to be clarified, which might limit the clinical value of the proposed prediction models.

1. https://github.com/54rabbits/CESM_Radiomics_Model.git

## Data Availability Statement

The original contributions presented in the study are included in the article/**Supplementary Material**. Further inquiries can be directed to the corresponding authors.

## Author Contributions

XW and YG were responsible for literature research, experimental studies, statistical analysis, and drafting of the article. YSa was responsible for experimental studies and statistical analysis and helped to draft the article. YPS, YC, and WJ were responsible for literature research and clinical studies and helped to draft the article. XL, YL, DX, and YSu contributed to the clinical studies and helped to draft the article. HY was responsible for literature research and statistical analysis and helped to draft the article. All authors contributed to the article and approved the submitted version.

## Funding

The study was supported by Major Science and Technology Projects in Tianjin (18ZXZNSY00240), Shandong Provincial Natural Science Foundation (No. ZR202102210508), Shandong Medical and Health Science and Technology Development Project (No. 202004081034), Special fund for clinical research of Wu Jieping Medical Foundation (No. 320.6750.2020-20-4), and Yantai Science and Technology Innovation Development Plan Project (No. 2021YD007, 2021YD005).

## Conflict of Interest

The authors declare that the research was conducted in the absence of any commercial or financial relationships that could be construed as a potential conflict of interest.

## Publisher’s Note

All claims expressed in this article are solely those of the authors and do not necessarily represent those of their affiliated organizations, or those of the publisher, the editors and the reviewers. Any product that may be evaluated in this article, or claim that may be made by its manufacturer, is not guaranteed or endorsed by the publisher.

## Abbreviations

NSLN, non-sentinel lymph node; SLN, sentinel lymph node; ALND, axillary lymph node dissection; CESM, contrast-enhanced spectral mammography; LASSO, least absolute shrinkage and selection operator; ANOVA, analysis of variance; LVI, lymphovascular invasion; AUC, area under the receiver operating characteristic curve; CI, confidence interval; ALN, axillary lymph node; HER-2, human epidermal growth factor receptor 2; CC, craniocaudal; MLO, mediolateral oblique; DICOM, Digital Imaging and Communications in Medicine; DCA, decision curve analysis; ROI, region of interest; PPV, positive predictive value; NPV, negative predictive value; MSE, mean square error; CNN, convolutional neural network; ROC, receiver operating characteristic.
