# Supplementary material for: Contrast-Enhanced Spectral Mammography-Based Prediction of Non-Sentinel Lymph Node Metastasis and Axillary Tumor Burden in Patients With Breast Cancer
Source: Front Oncol. 2022 May 6;12:823897. doi: 10.3389/fonc.2022.823897 (PMC9125761; doi:10.3389/fonc.2022.823897)
Supplement: Supplementary file 1 [file DataSheet_1.docx]

Supplementary Material

# Supplementary Figures and Tables

## Supplementary Figures

##
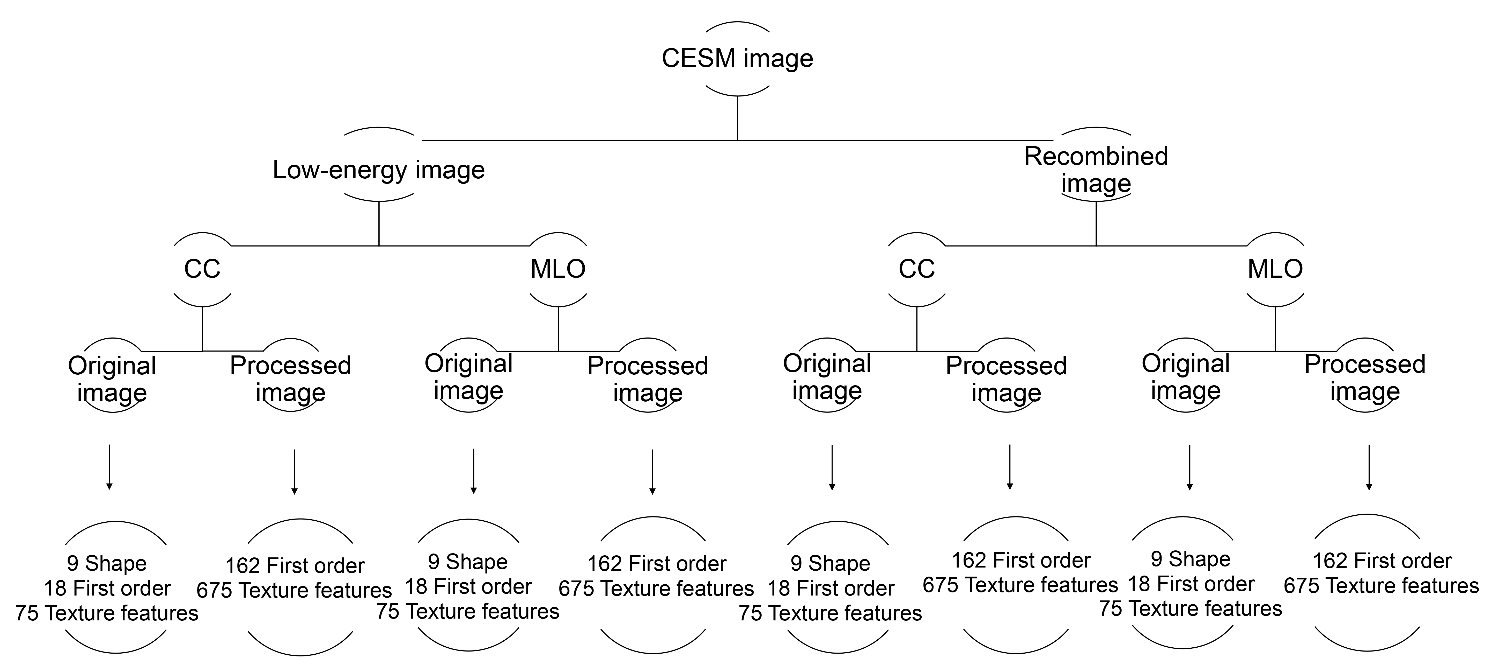


**Supplementary Figure 1**: All extracted radiomics features from CESM image.


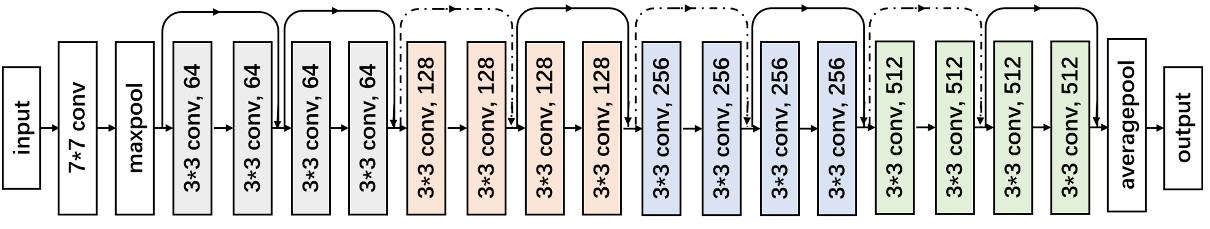


**Supplementary** **Figure 2:** The structure of ResNet-18 [1].


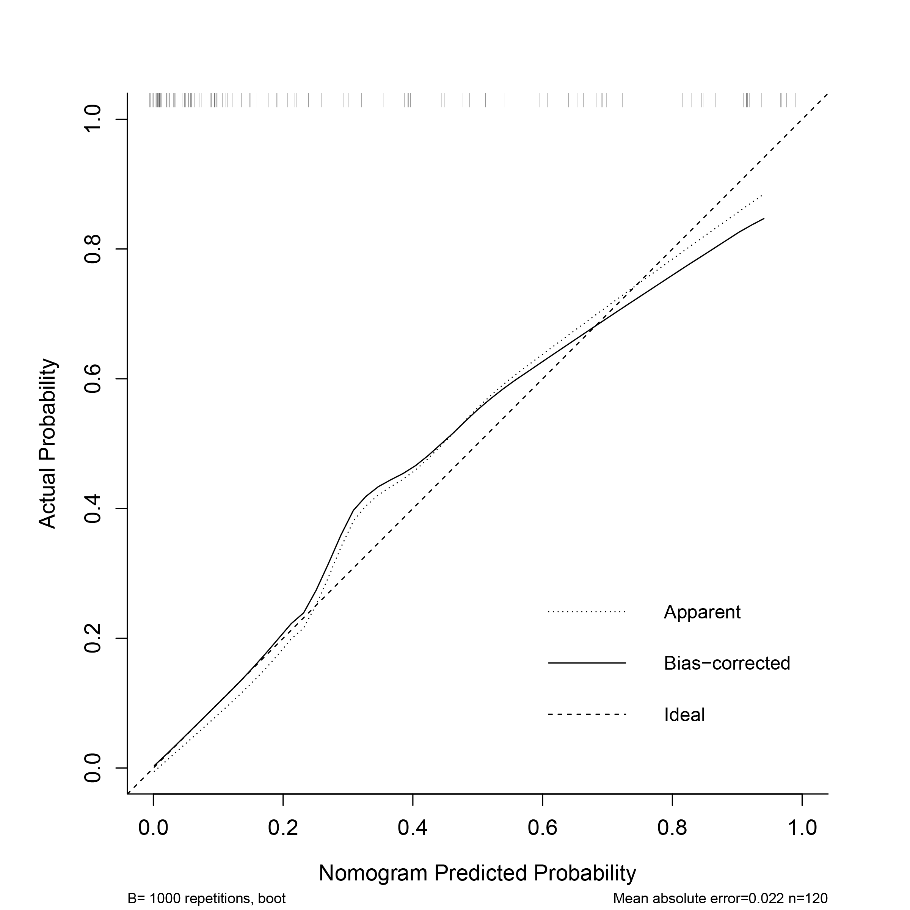


**Supplementary Figure 3:** The calibration plot for the nomogram. The x-axis is the nomogram predicted probability and y-axis is the actual probability of NSLN metastasis. The perfect prediction of the nomogram would correspond to the diagonal dotted line. The black solid line represents the nomogram performance.

**
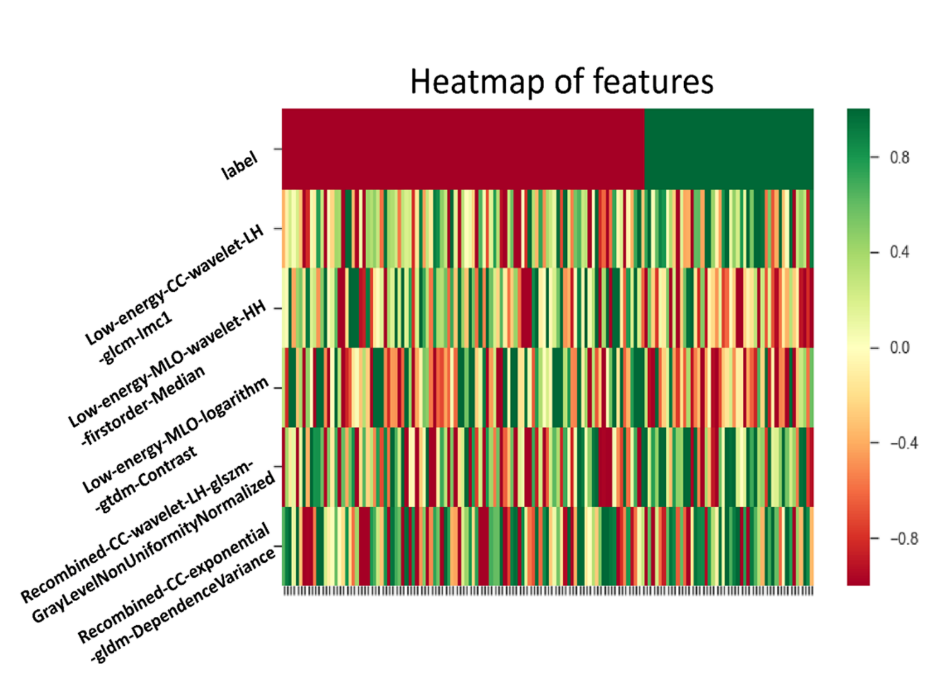
**

**Supplementary Figure 4.** Heatmap showed the distribution of quantitative radiomics features.

He K, Zhang X, Ren S, Sun J. (2016). “Deep Residual Learning for Image Recognition”, in: 2016 IEEE Conference on Computer Vision and Pattern Recognition (CVPR). Las Vegas, NV, USA: IEEE.
